# Urticaria Treated by Adrenalin

**Published:** 1913-11

**Authors:** 


Urticaria Treated by Adrenalin. Swann of New York, *Boston M. and S. Jour.*, May 1, 1913, treated eight cases of urticaria with hypodermics of adrenalin solution, standardized at half a milligram for an adult, repeated after ten minutes. In all cases the lesions disappeared after the second dose, even within a few minutes. He intimates that the cure was not permanent in all, but had no opportunity for learning if repeated injections would effect an actual cure.

